# Cost-effectiveness analysis of neonatal hearing screening program in china: should universal screening be prioritized?

**DOI:** 10.1186/1472-6963-12-97

**Published:** 2012-04-17

**Authors:** Li-Hui Huang, Luo Zhang, Ruo-Yan Gai Tobe, Fang-Hua Qi, Long Sun, Yue Teng, Qing-Lin Ke, Fei Mai, Xue-Feng Zhang, Mei Zhang, Ru-Lan Yang, Lin Tu, Hong-Hui Li, Yan-Qing Gu, Sai-Nan Xu, Xiao-Yan Yue, Xiao-Dong Li, Bei-Er Qi, Xiao-Huan Cheng, Wei Tang, Ling-Zhong Xu, De-Min Han

**Affiliations:** 1 Beijing Tongren Hospital, Capital Medical University, Beijing, China; 2 Beijing Institute of Otolaryngology, Key Laboratory of Otolaryngology Head and Neck Surgery (Capital Medical University), Ministry of Education, China, 17 Hou-gou Lane, Chong-nei Street, Beijing 100005, China; 3 Graduate School of Medicine, The University of Tokyo, Tokyo, Japan; 4 Tokyo University Hospital, Tokyo, Japan; 5 Institute of Social Medicine and Health Services Management, School of Public Health, Shandong University, Wen-hua-xi Road No.44, Jinan City, Shandong Province 250012, China; 6 Guangdong Province Maternal and Child Health Hospital, Guangzhou, Guangdong, China; 7 Beijing Haidian District Maternal and Child Heath Hospital, Beijing, China; 8 Hebei Province Langfang City Maternal and Child Health Hospital, Langfang, Hebei, China; 9 Henan Province Anyang City Maternal and Child Heath Hospital, Anyang, Henan, China; 10 Jiangxi Province Jiujiang City Maternal and Child Health Hospital, Jiujiang, Jiangxi, China; 11 Guangxi Province Liuzhou City Maternal and Child Heath Hospital, Liuzhou, Guangxi, China; 12 Beijing Shangdi Hospital, Beijing, China; 13 The Third People's Hospital of Wenzhou City, Wenzhou, Zhejiang, China; 14 Hebei Province Chengde City Maternal and Child Health Hospital, Chengde, Hebei, China; 15 Hebei Province Tangshan City Maternal and Child Health Hospital, Tangshan, Hebei, China

## Abstract

**Background:**

Neonatal hearing screening (NHS) has been routinely offered as a vital component of early childhood care in developed countries, whereas such a screening program is still at the pilot or preliminary stage as regards its nationwide implementation in developing countries. To provide significant evidence for health policy making in China, this study aims to determine the cost-effectiveness of NHS program implementation in case of eight provinces of China.

**Methods:**

A cost-effectiveness model was conducted and all neonates annually born from 2007 to 2009 in eight provinces of China were simulated in this model. The model parameters were estimated from the established databases in the general hospitals or maternal and child health hospitals of these eight provinces, supplemented from the published literature. The model estimated changes in program implementation costs, disability-adjusted life years (DALYs), average cost-effectiveness ratio (ACER), and incremental cost-effectiveness ratio (ICER) for universal screening compared to targeted screening in eight provinces.

**Results and discussion:**

A multivariate sensitivity analysis was performed to determine uncertainty in health effect estimates and cost-effectiveness ratios using a probabilistic modeling technique. Targeted strategy trended to be cost-effective in Guangxi, Jiangxi, Henan, Guangdong, Zhejiang, Hebei, Shandong, and Beijing from the level of 9%, 9%, 8%, 4%, 3%, 7%, 5%, and 2%, respectively; while universal strategy trended to be cost-effective in those provinces from the level of 70%, 70%, 48%, 10%, 8%, 28%, 15%, 4%, respectively. This study showed although there was a huge disparity in the implementation of the NHS program in the surveyed provinces, both universal strategy and targeted strategy showed cost-effectiveness in those relatively developed provinces, while neither of the screening strategy showed cost-effectiveness in those relatively developing provinces. This study also showed that both strategies especially universal strategy achieve a good economic effect in the long term costs.

**Conclusions:**

Universal screening might be considered as the prioritized implementation goal especially in those relatively developed provinces of China as it provides the best health and economic effects, while targeted screening might be temporarily more realistic than universal screening in those relatively developing provinces of China.

## Background

As adequate auditory stimulation in early childhood is fundamental for optimal speech and language development as well as for the acquisition of literacy skills, early hearing detection and interventions for deaf children are essential [1,2]. A failure to undertake early hearing detection and intervention within the first year of life for permanent congenital and early-onset hearing impairment (PCEHI) might lead to severe and irreversible impairment in language acquisition and speech development in early life and poor educational and occupational performance in adulthood [3-7]. However, compared to those major disease set out in the Millennium Development Goals, PCEHI, as a non-fatal but life-long disease, has been neglected particularly in developing countries [8-10].

Neonatal hearing screening (NHS) program has made a rapid development in recent years. The benefit of NHS is that it allows most PCEHI to be detected early enough for optimal intervention [11,12]. NHS has been routinely offered as a vital component of early childhood care in developed countries, whereas such a screening program is still at the pilot or preliminary stage as regards its nationwide implementation in developing countries where hearing care services, such as the provision of hearing aids, only cover approximately 1% of all the population [13].

Cost-effectiveness analysis (CEA) has been used as a tool to prioritize scarce health resources, which allows the comparison of various intervention options [14,15]. Although CEA is currently the cornerstone of health investment, it might be the only one approach implemented in the developing countries in Africa and Asia to evaluate selected interventions for hearing impairment [16]. The prospects of any immediate action to implement NHS programs are still uncertain in most of developing countries, since the current approach to global disease prioritization and health resource allocation requires vital data in an environment where funds to invest are always scarce.

In China, the largest developing country in the world, the Ministry of Health has recently decided to address the issue of non-fatal congenital diseases in neonatal health care which have until now been neglected but have a considerable impact on the individual, family and society. There is now a political commitment to scale-up a hospital-based neonatal hearing screening program, which will include expanded screening, diagnosis and intervention services [17]. A key policy question on how to implement the program under a system of decentralized health financing and authority at the provincial level has been raised. Therefore, this study aims to determine cost-effectiveness of the NHS program implementation, in case of eight provinces of China, in order to support evidence-based national policy making in China.

## Methods

### Cost-effectiveness model

**Figure 1:**
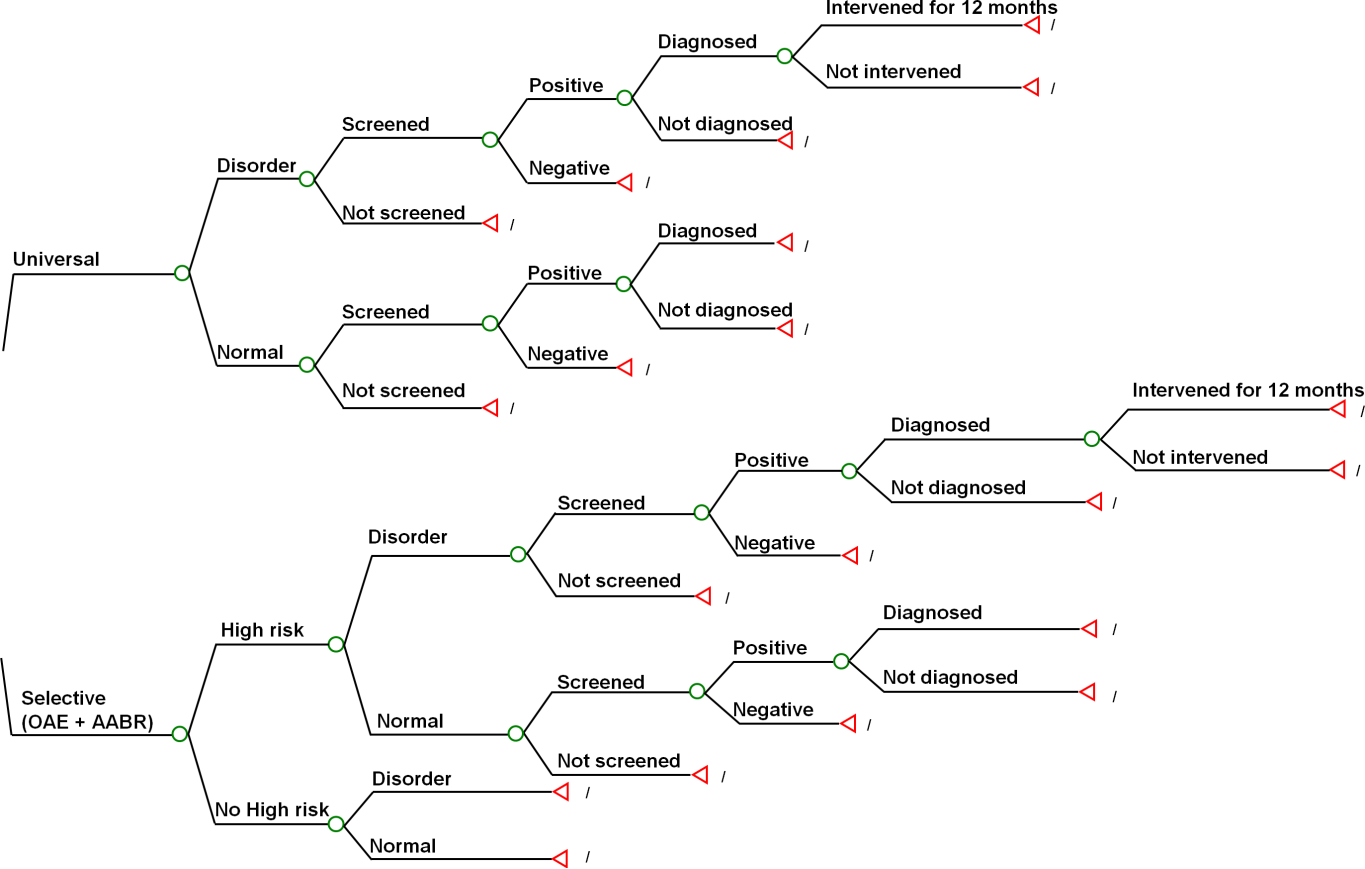
**The tree model for cost-effectiveness of two screening strategies (universal screening and targeted screening)**.

In the present study, we modeled the cost-effectiveness of two screening programs: universal screening which covers all live births, and targeted screening which targets those with one or more risk factors (Figure 1). Universal NHS detects infants with the disorder who have no known risk factors associated with PCEHI, which accounts for approximately 50% of PCEHI cases [18,19]. The targeted screening of newborns with risk factors for hearing impairment is one alternative to universal NHS. Although the universal strategy has been widely recommended as it can detect more cases, recently Joint Committee on Infant Hearing (JCIH) suggested that targeted screening might be more appropriate for developing countries because of cost considerations [20].

**Figure 2:**
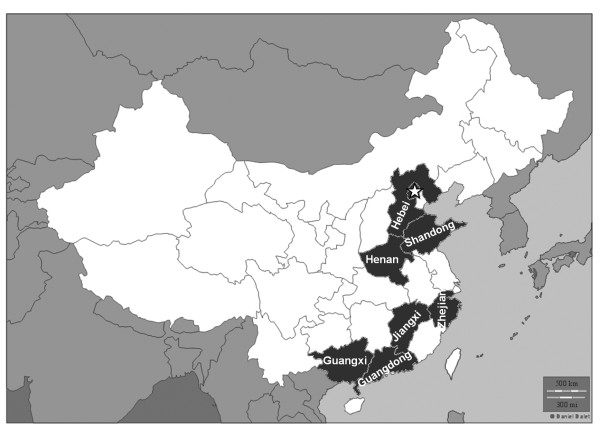
**The map of study sites including Beijing (☆), Shandong, Hebei, Henan, Jiangxi, Zhejiang, Guangxi and Guangdong**.

A natural history model of all infants with and without hearing impairments was developed to characterize the process of screening, diagnosis and interventions through all possible stages. All neonates annually born in eight provinces (Beijing, Shandong, Hebei, Henan, Jiangxi, Guangxi, Guangdong, and Zhejiang) of China from 2007 to 2009 were simulated (Figure 2). In these 8 provinces of China, Beijing, Shandong, Hebei, Guangdong, and Zhejiang are relatively developed provinces, whereas Henan, Jiangxi, and Guangxi are relatively developing provinces. The model determined the proportion of infants with PCEHI in the simulated cohort who would potentially benefited from the NHS program, where detection by a process of screening and diagnosis occurs before 6 months and intervention before 12 months.

### Probability parameters

Depending on the program coverage and screening strategies, neonates could either receive or not receive screening. Neonates screened consisted of false-positive and false-negative cases, and those with a positive result are referred to the diagnosis center. With the probability of drop-out from the diagnosis center and an inaccessibility of interventions, referred neonates might not be either diagnosed or receive interventions. In the final instance, infants with the disorder would have treated or untreated health outcomes, depending on whether they received screening, diagnosis and interventions.

In this model, probability parameters potentially affecting cost-effectiveness are the proportion of infants with one or more high risks, the prevalence of PCEHI in the general population and high-risk population, the sensitivity, specificity, and coverage of the screening program which shows neonates screened among those targeted by the screening program (general population or high-risk population), diagnosis rate which shows those diagnosed before 6 months among those referred after the screening, and intervention rate which shows infants fitted with hearing aids or cochlear implants and that have started the rehabilitation course before 12 months of age among those with the disorder as detected by the diagnosis process (Table 1). The coverage, diagnosis rate and intervention rate were calculated based on data in the newly established database in the study sites, while others were acquired from literature review.

**Table 1:** Parameter values and plausible ranges for probability variables used in the baseline and sensitivity analysis

Items	Baseline	Range for sensitivity analysis	References
High-risk infants	7%	6-8%	[21]

Prevalence in all live-born	0.30%	0.2-0.4%	[22] [23] [24] [25] [26]

Prevalence in high risk infants	3.00%	1.15-3.59%	[27] [28] [29] [30] [31] [32]

Sensitivity	95%	90-100%	[17] [32] [33]

Specificity	95%	90-100%	[18] [34] [35]

Coverage	60%	15-99%	[36] (Primary data)

Diagnosis rate	50%	20-95%	[36] (Primary data)

Intervention rate	50%	10-95%	[36] (Primary data)

### Costs for the program implementation

We followed the guideline developed by the World Health Organization (WHO) [37] to estimate costs for the program implementation. These included capital costs (*e.g. *office buildings, furniture and equipment), recurrent costs such as the salaries of the personnel, materials and supplies, utilities, equipment maintenance, database management, training and transport, and patient-level costs which were composed of the registration fee, screening tests, diagnosis tests, drugs, treatment with a hearing aid, treatment with a cochlear implant, rehabilitation courses, and transportation charges for diagnostic procedures and treatments. The capital investments of office buildings, furniture and equipment were annualized by depreciation, for which the life spans were standardized by Ministry of Finance of the People's Republic of China and Ministry of Health of the People's Republic of China [38] Table 2 summarized cost estimates for cost-effectiveness analysis. Data for the cost estimates came from the annual financial report of screening facilities, diagnosis centers and rehabilitation facilities in the surveyed provinces, with the cooperation of experts in hospital accounting. Costs were first calculated in RMB, the Chinese currency, with the costs across different years being adjusted in accordance with the price level in 2009 based on the GDP deflator. They were then converted into international dollars (Int$), by dividing Purchasing Power Parities [39].

**Table 2:** Parameter values and plausible ranges for cost estimates per case used in the baseline and sensitivity analysis (Int $)

Items	Hearing screening method	Baseline	Ranges for sensitivity analysis	
			
			Minimum	Maximum
Screening				

Program costs				

Capital costs	OAE	240,813,700	170,712,610	307,291,540

	OAE+AABR	429,951,870	322,027,570	559,461,040

Recurrent costs	OAE	150	100	200

	OAE+AABR	230	130	360

Patient costs		30	10	50

Diagnosis				

Program costs				

Capital costs		15,950,600	11,014,773	21,467,600

Recurrent costs		240	130	360

Patient costs		170	90	310

Intervention				

Program costs				

Capital costs		13,829,240	12,430,260	15,554,540

Recurrent costs		290	200	440

Patient costs		22,690	18,860	29,140

Abbreviation: Otoacoustic emission (OAE); Automated auditory brainstem response (AABR)

Apart from the costs of program implementation for estimates of cost-effectiveness, it is reasonable to assume (and thus estimate) that money would be saved if early detection and intervention was given. Early detection and intervention may bring expenditure on special education and rehabilitation reduction, social and medical services support decrease and incomes increase. However, there was no report on the long-term costs of the disorder from the published literature in China. In our model, we preliminarily attempted to estimate the potential long-term costs saving from the use of the NHS program, including medical services, special education and rehabilitation. It was assumed that improved language outcomes would result in a 10% decrease in special education costs and a 75% decrease in vocational rehabilitation costs [40,41]. It was estimated based on data from hospitals, disabled people's federations [42] the provincial health agency and the provincial education agency. Table 3 presented a summary of the long-term costs saving.

**Table 3:** Summary of estimates for long-term costs saving

Items	long-term costs saving (Int $)	
	
	Untreated children with PCEHI	Treated Children with PCEHI
Medical services		

For cases to fit hearing aid	1,960 (1,740-2,180)	1,960 (1,740-2,180)

For cases to fit cochlear implant	40,000 (38,000-42,500)	40,000 (38,000-42,500)

Special education (9 years compulsory education)	42,300 (40,500-45,900)	38,070 (36,450-41,310)

Rehabilitation (Up to 15 years)	82,400 (75,200-88,400)	20,600 (18,800-22,100)

Total	166,660 (155,440-178,980)	100,630 (60,790-108,090)

### Data collection

Data collection in the field included two components: costs related to the screening program which were acquired from general hospitals and maternal and child health hospitals providing screening and diagnosis services, and rehabilitation facilities, and transition probability parameters including the coverage of screening program, the diagnosis rate and intervention rate which were calculated based on data from the database established at the provincial level. A multiple of three parameters shows the proportion of infants with PCEHI potentially benefiting from the screening program among the targeted population (all or high-risk infants), in this study, calculated as the proportion of the benefit population [43].

After permission to use the database by the district health agencies, the data of the province were acquired. For the costs estimate, all facilities providing screening, diagnosis, and rehabilitation services were visited in selected districts and the annual financial report was analyzed by an expert in hospital accounting.

### Estimates of health effects

In this study, population health is expressed as the number of Disability-Adjusted Life Years (DALYs) averted as a result of the screening program. DALYs lost due to PCEHI were calculated as the sum of Years Lost due to Disability (YLDs), as it is a non-fatal disorder, and the time period is life-long. Disability weights for adult-onset hearing impairment were adopted in the estimation, 0.216 for untreated disorders and 0.168 for treated disorders, respectively [44]. This was done because there was no evaluation for infants and children, and lifetime coverage rather than childhood coverage was thought to be able to minimize the discrepancy in the long term.

### Cost-effectiveness analysis

The cost-effectiveness of a given intervention is typically expressed as costs per unit of effectiveness, with costs measured in monetary terms and effectiveness measured in health metrics terms. In this study, the average cost-effectiveness ratio (ACER) is calculated for each screening strategy by determining the cost for the program's implementation (except long-term costs saving) and total health effects in terms of DALYs averted. Incremental cost-effectiveness ratio (ICER) shows the absolute value of resources necessary to move to the next option. We calculated ICER for different screening strategies by dividing the incremental costs by the incremental health effects, in order to determine the priority of purchasing these services at different budgetary levels.

A multivariate sensitivity analysis was performed to determine uncertainty in health effect estimates and cost-effectiveness ratios, using a probabilistic modeling technique, known as Monte Carlo simulation. The simulation associating the estimates of costs and health effects as described above with each of the transition probability parameters was performed on one parameter at a time allowing for the input of extreme values while keeping the other parameters fixed at the their baseline level. The model was evaluated on 1,000 trials. It showed that any variation in the input parameter might result in a change of preference of strategies compared to the baseline result.

## Results

### Benefit population of the current neonatal hearing screening program

Demographic and socioeconomic information and the indicators of the program implementation including coverage rate, diagnosis rate and intervention rate in the six provinces were presented in Table 4. The three variables (coverage rate, diagnosis rate and intervention rate) determined the total number of deaf infants finally receiving early interventions and benefiting from the screening program. The benefit population referred to the proportion of infants with the disorder who finally received beneficial early hearing detection and intervention. There was a huge disparity in this figure between different provinces. In general, these figures in the developed eastern provinces were much higher.

**Table 4:** Basic information of study sites

Provinces	Beijing	Shandong	Hebei	Zhejiang	Guangdong	Henan	Jiangxi	Guangxi
Population^a^	16,330,000	93,670,000	69,430,000	50,600,000	94,490,000	93,600,000	4,368,000	47,680,000
Birth rate (per 1,000)^a^	8.32	11.11	13.33	11.26	11.96	11.26	13.86	14.19
No. of live births per year^a^	135,866	1,040,674	925,502	1,053,936	1,130,100	1,053,936	605,405	676,579
GDP per capita (Int$)^b^	16,644.25	7,951.80	5,684.11	10,698.20	9,479.99	4,578.86	3,612.58	3,590.28
Life expectancy^a^	76.1	73.92	72.54	74.7	73.27	71.54	68.95	71.29
Development status^c^	Developed	Developed	Developed	Developed	Developed	Moderately developed	Moderately developed	Less developed
No. of screening facilities^a^	548	1,402	1,296	722	1,154	1,341	602	553
No. of diagnosis centers^a^	6	17	11	11	21	17	11	14
No. of rehabilitation facilities^a^	12	14	8	8	15	10	6	5
Coverage rate^d^	97.8%	83.3%	91.3%	83.3%	97.0%	24.5%	30.5%	50.2%
Diagnosis rate^d^	97.4%	68.3%	60.0%	60.0%	75.0%	30.2%	48.2%	21.4%
Intervention rate^d^	77.1%	72.5%	76.1%	70.0%	75.0%	23.8%	33.3%	23.9%
Proportion of benefit population	73.4%	41.3%	41.7%	35.0%	54.6%	1.8%	4.9%	2.6%

Data source:

^a^: Ministry of Health of the People's Republic of China [17].

^b^: International Monetary Fund [39].

^c^: Ministry of Health of the People's Republic of China [35].

^d^: Provincial data

### Cost-effectiveness of different strategies in eight provinces

Table 5 shows implementation costs, DALYs averted, and cost-effectiveness of different NHS strategies in eight provinces. Based on GDP per capita in each province and baseline of transition probability parameters, universal strategy shows cost-effectiveness in Guangdong, Shandong, and Beijing, and targeted strategy shows cost-effectiveness in Zhejiang and Hebei, while neither of the screening strategy shows cost-effectiveness in Guangxi, Jiangxi and Henan.

**Table 5:** Implementation costs, health effects and cost-effectiveness of different NHS strategies in eight provinces

Items	Guangxi	Jiangxi	Henan	Guangdong	Zhejiang	Hebei	Shandong	Beijing
Total costs (Int $)								
Universal strategy	10,498,335	23,063,105	9,121,907	39,077,961	17,144,588	33,472,076	32,326,020	4,014,771
Targeted strategy	1,416,185	2,272,838	2,403,868	6,774,350	2,022,018	4,554,258	4,880,764	1,094,184
DALY averted								
Universal strategy	35	206	78	3,508	278	1,499	1,533	292
Targeted strategy	17	101	38	1,719	136	735	751	143
ACER								
Universal strategy	299,952	111,957	116,948	11,140	61,671	22,330	21,087	13,749
Targeted strategy	83,305	22,503	63,260	3,941	14,868	6,196	6,499	7,652
Reference (3 times GDP per capita)	10,771	10,838	13,737	28,440	32,095	17,052	23,855	49,933
ICER								
Universal strategy	504,564	198,003	167,951	18,057	106,497	37,851	35,096	19,601
Targeted strategy	83,305	22,503	63,260	3,941	14,868	6,196	6,499	7,652

**Figure 3:**
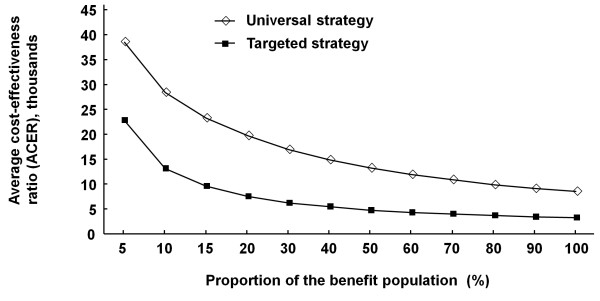
**The relationship of the average cost-effectiveness ratio (ACER) and the proportion of benefit population for two screening strategies (universal screening and targeted screening)**.

Multivariate sensitivity analyses were performed for transition probability parameters to determine the robustness of the model. The results suggested the variables whose range of uncertainty had a great impact on the cost-effectiveness of the screening strategies were the program coverage, diagnosis rate, and intervention rate. Figure 3 showed that the increasing proportion of the benefit population, which was estimated by multiplying the three variables, reduced ACER of different strategies and gradually helped both reach better cost-effectiveness. Targeted strategy trended to be cost-effective in Guangxi, Jiangxi, Henan, Guangdong, Zhejiang, Hebei, Shandong, and Beijing from the level of 9%, 9%, 8%, 4%, 3%, 7%, 5%, and 2%, respectively; while universal strategy trended to be cost-effective in those provinces from the level of 70%, 70%, 48%, 10%, 8%, 28%, 15%, 4%, respectively.

### Economic effects on long-term costs saving

Costs would be saved if early detection and intervention was given. In our model, universal strategy and targeted strategy led to 214,024,820 and 104,872,740 Int$ of long-term costs saving in total which are approximately equivalent to 0.14% and 0.07% of the annual health expenditure [36].

**Figure 4:**
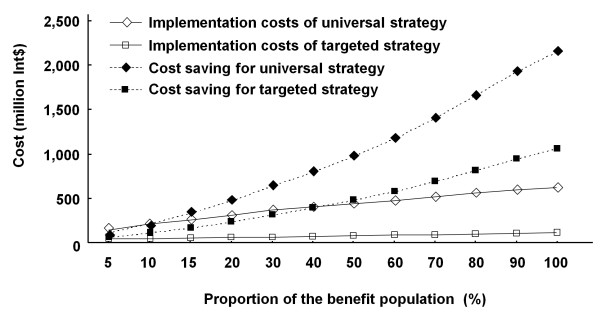
**The relationship of the long-term costs saving or implementation costs of two screening strategies (universal screening and targeted screening) and the proportion of benefit population**.

At the baseline, both strategies achieve cost savings which were greater than implementation costs. When the proportion of benefit population expanded, the effect of these screening strategies on the long-term costs saving become more and more significant, especially those of universal strategies, exceeding the total costs of the screening program implementation, suggesting a good economic effect in the long term (Figure 4).

## Discussion

WHO has recommended the implementation of NHS program in member states, particularly in developing countries, based on the experiences and contributions of leading experts from various world regions and across relevant disciplines [45]. This study found there was a huge disparity in the implementation of the NHS program in the surveyed provinces. In general, those developed provinces had much higher program coverage, diagnosis rates and intervention rates, leading to a larger benefit population and helping the NHS program achieve better cost-effectiveness. In fact, the NHS program had been adopted several years in some developed provinces in China with financial and administrative supports by local health government [46]. Under the decentralization of health financing, the developed provinces have a stronger financial capacity to invest in health programs [47]. This difference in implementation and in underlying financial factors has also been reflected in the national plan. Regarding the accessibility of the screening, diagnosis and intervention services, the short-term goal is to achieve a coverage rate of 80% in the eastern provinces (the most developed region of China), 40% in the moderate provinces, and 30% in the western provinces (the least developed region of China) by 2012, and to increase these figures to 90%, 60% and 50%, respectively, by 2015 [17].

Can the national plan help the NHS program achieve its goal with good cost-effectiveness, health and long-term economic effects, at both the national and the regional level? The goal of the NHS program in China is to establish a nationwide hospital-based universal program and to continuously expand diagnosis and intervention services [17]. The rationale for implementing a universal strategy is that it can detect more deaf infants, providing a greater opportunity for them to experience normal language development, while also providing overall benefits in terms of the reduction in disability and the improvement in health and well-being of the Chinese population. As the program's implementation varies by different socioeconomic development status, a sufficiently high coverage rate, diagnosis rate and intervention rate ensure that the universal strategy achieves good cost-effectiveness and the relevant health and economic effects in Beijing, Shandong and Hebei; but in the other three provinces Henan, Jiangxi and Guangxi, where these three indicators were low, targeted strategies tend to be more feasible, similar to previous policy suggestions that have been advanced in relation to developing countries [20,48]. Meanwhile, a pilot survey on context-specific risk factors for PCEHI in diverse settings should be implemented to facilitate the targeted strategy.

The study also attempted to estimate the long-term economic effect of the NHS program in China. As the available data and evidence is limited and this is a very preliminary approach, only costs saving in rehabilitation and special education up to 15 years of age (at which children finish their compulsory education) were counted, potentially leading to an underestimate of the economic effect. Even so, the results showed that at the baseline, the costs saved already exceeded the implementation costs in the targeted strategies; as the proportion of the benefit population expends, the economic effect would be more and more significant, especially in relation to universal strategies. Such results strongly support the implementation of the NHS program and suggest potentially greater benefits of adopting universal strategies in China.

Our study was restricted by the limited availability of data and evidence. First, globally, there was no strong evidence based on randomized controlled trials for the effectiveness of NHS on deaf infants' language development, and consequently, no evidence as regards the evaluation of their psychological and educational-related outcomes. The impact of early intervention on long-term cost saving remains unknown. Moreover, in China, there has been no previous nationwide population-based study to survey the epidemiological status of PCEHI. Data on the prevalence used in this study were therefore derived from a crude estimation based on the number of the hearing disabled population and a few regional surveys. All that needs to be researched in future. Last, due to the unavailability of child-onset disability weights for hearing loss, we attempted to use adult-onset disability weights instead into the economic evaluation. It may underestimate the health effects of NHS as early detection and intervention leads to much more benefits on children's language development.

## Conclusions

In conclusion, universal strategy can be considered as the ultimate implementation goal as it provides the best health and economic effects. A universal strategy is feasible in provinces where screening, diagnosis and intervention services are good enough to benefit sufficient proportion of the deaf children. In the other regions, a targeted strategy is temporarily more realistic than a universal strategy; however, related services still need to be scaled up to cover the targeted population as much as possible. The reference data from this study thus are expected to be of particular benefit in terms of the 'rolling out' of the national plan.

## Abbreviations

PCEHI: Permanent congenital and early-onset hearing impairment; NHS: Neonatal hearing screening; CEA: Cost-effectiveness analysis; DALYs: Disability-Adjusted Life Years; YLDs: Years Lost due to Disability; ACER: Average cost-effectiveness ratio; ICER: Incremental cost-effectiveness ratio.

## Competing interests

The authors declare that they have no competing interests.

## Authors' contributions

WT, LZX and DMH designed the study. LHH, LZ, RYGT, FHQ, LS, YT, QLK, FM, XFZ, MZ, RLY, LT, HHL, YQG, SNX, XYY, XDL, BEQ and XHC collected data for the study. LHH, LZ, RYGT and FHQ analyzed the data and contributed to the writing of the paper.

## Pre-publication history

The pre-publication history for this paper can be accessed here:

http://www.biomedcentral.com/1472-6963/12/97/prepub
